# Electronic and atomic structures of the Sr_3_Ir_4_Sn_13_ single crystal: A possible charge density wave material

**DOI:** 10.1038/srep40886

**Published:** 2017-01-20

**Authors:** H.-T. Wang, M. K. Srivastava, C.-C. Wu, S.-H. Hsieh, Y.-F. Wang, Y.-C. Shao, Y.-H. Liang, C.-H. Du, J.-W. Chiou, C.-M. Cheng, J.-L. Chen, C.-W. Pao, J.-F. Lee, C. N. Kuo, C. S. Lue, M.-K. Wu, W.-F. Pong

**Affiliations:** 1 Department of Physics, National Tsing Hua University, Hsinchu 300, Taiwan; 2 Department of Physics, Tamkang University, Tamsui 251, Taiwan; 3 Department of Applied Physics, National University of Kaohsiung, Kaohsiung 811, Taiwan; 4 National Synchrotron Radiation Research Center, Hsinchu 300, Taiwan; 5 Department of Physics, National Cheng Kung University, Tainan 700, Taiwan; 6 Institute of Physics, Academia Sinica, Taipei 115, Taiwan

## Abstract

X-ray scattering (XRS), x-ray absorption near-edge structure (XANES) and extended x-ray absorption fine structure (EXAFS) spectroscopic techniques were used to study the electronic and atomic structures of the high-quality Sr_3_Ir_4_Sn_13_ (SIS) single crystal below and above the transition temperature (T^*^ ≈ 147 K). The evolution of a series of modulated satellite peaks below the transition temperature in the XRS experiment indicated the formation of a possible charge density wave (CDW) in the (110) plane. The EXAFS phase derivative analysis supports the CDW-like formation by revealing different bond distances [Sn_1(2)_-Sn_2_] below and above T^*^ in the (110) plane. XANES spectra at the Ir *L*_3_-edge and Sn *K*-edge demonstrated an increase (decrease) in the unoccupied (occupied) density of Ir 5*d*-derived states and a nearly constant density of Sn 5*p*-derived states at temperatures T < T^*^ in the (110) plane. These observations clearly suggest that the Ir 5*d*-derived states are closely related to the anomalous resistivity transition. Accordingly, a close relationship exists between local electronic and atomic structures and the CDW-like phase in the SIS single crystal.

Transition-metal dichalcogenides are well known for their many fascinating physical properties, including superconductivity, charge or spin density waves and strong electron-phonon coupling^1^,^2^,^3^,^4^,^5^,^6^. Layered transition-metal compounds, such as *TX*_2_ (*T* = transition metal and *X* = chalcogen), typically exhibit superconducting and charge density wave (CDW) properties, which are supposed to coexist and compete in *TX*_2_ materials^2^,^3^,^4^,^6^. For example, the 4*d* transition-metal layered structure of NbSe_2_ exhibits superconductivity at the critical temperature T_C_ ~ 7.2 K and at the CDW transition temperature T_CDW _~ 33.5 K^3^,^4^,^6^. Additionally, some 5*d* transition-metal systems have been found to exhibit both superconductivity and CDW features^2^.

The family of ternary stannide materials with an *R*_3_*T*_4_*Sn*_13_ stoichiometry, where *R* = La, Sr or Ca and *T* = Ir, Rh or Co, have recently been studied owing to their interesting characteristics, such as superconductivity, heavy fermions, complex magnetism, electricity and thermoelectric properties^7^,^8^,^9^,^10^,^11^,^12^,^13^,^14^,^15^,^16^,^17^,^18^,^19^,^20^,^21^,^22^,^23^,^24^,^25^. Superconducting properties of the Sr_3_Ir_4_Sn_13_ (SIS) and Sr_3_Rh_4_Sn_13_ (SRS) single crystals were recently observed with T_C_ ~ 5 K and ~4.7 K, respectively^7^,^9^,^16^,^20^. An interesting anomaly in the electrical resistivity of SIS and SRS compounds has been observed with transition temperatures T^*^ ~ 147 K and ~138 K, respectively, along with their superconducting feature^9^,^16^,^18^,^20^,^25^. The anomalous transition in resistivity varies with the doping concentration of Ca in SIS^16^. The observation of the anomalous resistivity transition in SIS has been understood, experimentally and theoretically, to involve a structural phase transition from the I phase (***a*** = 9.797 Å) to the I′ phase (***a*** = 19.595 Å) with a doubling of the lattice parameter^16^,^19^. D. A. Tompsett^19^ calculated the density of states (DOSs) of the constituent elements in SIS using the local-density approximation (LDA) at/near the Fermi surface and observed contributions from the Ir *d* and Sn *p* states and a negligible contribution from the Sr electronic states. He also observed a distortion with ***q*** = (0.5, 0.5, 0) at low temperature that may be linked to the formation of a CDW phase along ***q*** = (0.5, 0.5, 0). C. N. Kuo *et al*.^20^ have noted that the anomalous resistivity transition is associated with the reconstruction of the Fermi surface, which results in a decrease in the density of Ir 5*d* and Sn 5 *s* states at/near the Fermi surface of SIS. Therefore, the CDW property of the SIS single crystal has been speculated to possibly exist below T^*^ ~ 147 K (I′ phase), but whether it does remain a matter of debate^16^,^18^,^19^,^20^.

Numerous studies have used synchrotron radiation techniques to investigate CDW materials to elucidate the properties responsible for the CDW phenomena^1^,^3^,^4^,^5^,^6^,^21^,^25^,^26^,^27^,^28^,^29^,^30^,^31^. Temperature-dependent x-ray scattering (XRS) has been frequently used to verify that the series of satellite peaks associated with charge modulation are related to the CDW phenomena in certain orientations^21^,^29^,^30^,^31^. X-ray absorption spectroscopy studies have identified a change in the density of electronic states. This is associated with a phase transition in quasi one-dimensional (1D)-CDW materials^26^,^28^. Although previous studies have reported CDW phase formation at ~147 K in SIS, as mentioned above, conclusive experimental evidence on the details of the charge/electronic and atomic distortions related to the CDW phenomena is lacking. In particular, little is known about the contributions of the constituent elements, including Ir and Sn, to the phase transition that is significantly associated with the CDW property in a SIS single crystal.

In this study, a high-quality single crystal of SIS is examined in detail using XRS, x-ray absorption near-edge structure (XANES), extended x-ray absorption fine structure (EXAFS) and resistivity measurements at various temperatures. The temperature-dependent XRS results are consistent with those obtained for other well-established CDW systems, suggesting the CDW-like instability in the SIS system at the transition temperature. The results of EXAFS and XANES experiments reveal structural distortion at Sn sites and a decrease (an increase) in the DOSs of occupied (unoccupied) Ir 5*d*-derived states at/near the Fermi surface below the transition temperature in the (110) plane. These findings further support the possibility of CDW modulation at/below T^*^. Variation of the XANES feature at the Ir *L*_3_-edge clearly reveals that the Ir 5*d*-derived states, rather than the Sn 5*p*-derived states, at/near the Fermi surface are strongly associated with the anomalous resistivity transition in a SIS single crystal.

## Results and Discussion

**Figure 1:**
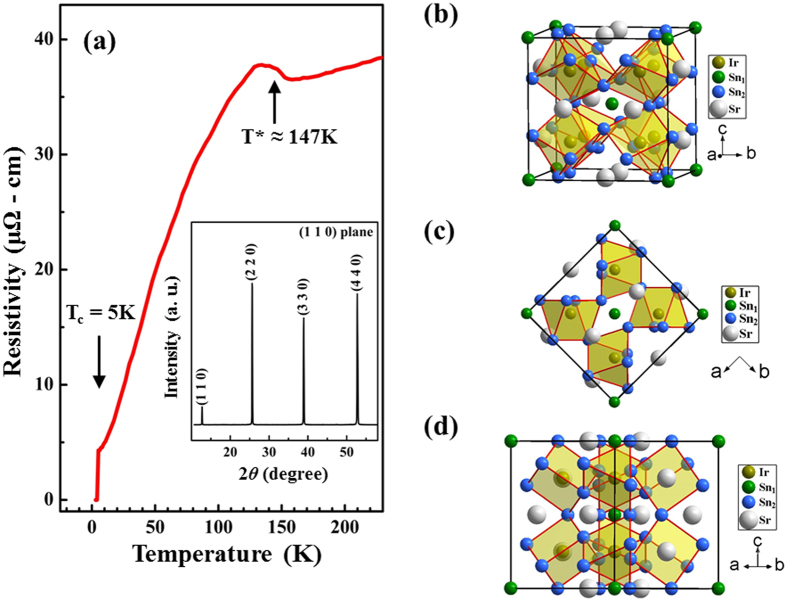
(a) Variation of electrical resistivity with temperature. Inset displays a room-temperature single-crystal XRD pattern, showing reflections from the (110) plane. **(b)** Cubic parent structure of SIS with partial view and atomic notation. Atomic arrangement in SIS, with the ***E***-field **(c)** parallel and **(d)** perpendicular to the (110) plane.

Figure 1(a) illustrates the temperature-dependent resistivity in the (110) plane of the sample surface. The inset in Fig. 1 presents the x-ray diffraction (XRD) pattern at room temperature, which reveals the favorability of the (110) texture in the SIS single crystal. The temperature-dependent resistivity exhibits an anomalous resistivity transition around T^*^ = 147 K and a superconducting phase transition at approximately 5 K. This finding is similar to that reported elsewhere and is understood as a CDW-like phase-induced structural transition and Fermi surface reconstruction at T^*^^16^,^18^,^20^,^25^. The SIS compound is also known to crystallize into different crystal structures below and above the transition temperature, T^*^^16^,^19^,^20^. At T > T^*^, the compound has a body-centered cubic structure in the I phase (*Pm*
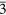
*n* space group, ***a*** = 9.7968 Å), which is converted into the I′ phase (*I*
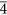
*3d* space group, ***a*** = 19.5947 Å) with a doubling of the lattice parameter below T^*^^16^. The crystal structure of SIS for T > T^*^ in Fig. 1(b) reveals single site occupancy of Sr and Ir and double occupancy sites for Sn atoms, i.e., Sn_1_ and Sn_2_ sites. Different colors have been used to visualize the constituting elements that form the crystal structure, as shown in Fig. 1(b–d). The Sn_1_ atom occupies the corner and body-centered positions of the cubic unit cell, forms edge-sharing Sn_1_(Sn_2_)_12_ icosahedra^7^,^16^, and is surrounded by 12 Sn_2_ atoms. Further, Sn_2_ atoms are bonded to the Ir atom in a trigonal prism fashion. In the I phase, all Sn_2_-Ir bond lengths are similar, whereas, in the I′ phase, the icosahedra at the Sn_2_ sites are distorted. The bond lengths of Sn_1_-Sn_2_ cease to be similar, and the Sn_2_ site occupies four different sites, Sn_21_, Sn_22_, Sn_23_ and Sn_24_, that form a complex structure. In Fig. 1(c,d), the atomic arrangements in the I phase of SIS are shown with the polarization, ***E***, of the electric field of synchrotron photons parallel and perpendicular to the (110) plane, respectively. From Fig. 1(c), the Sn_2_-Sn_2_ bonds (red lines) in the trigonal prisms are more ordered than those in Fig. 1(d). This structural difference results in a strong geometrical anisotropy, causing the physical properties to differ between the ***E***-field parallel and ***E***-field perpendicular to the (110) plane, as will be discussed below.

**Figure 2:**
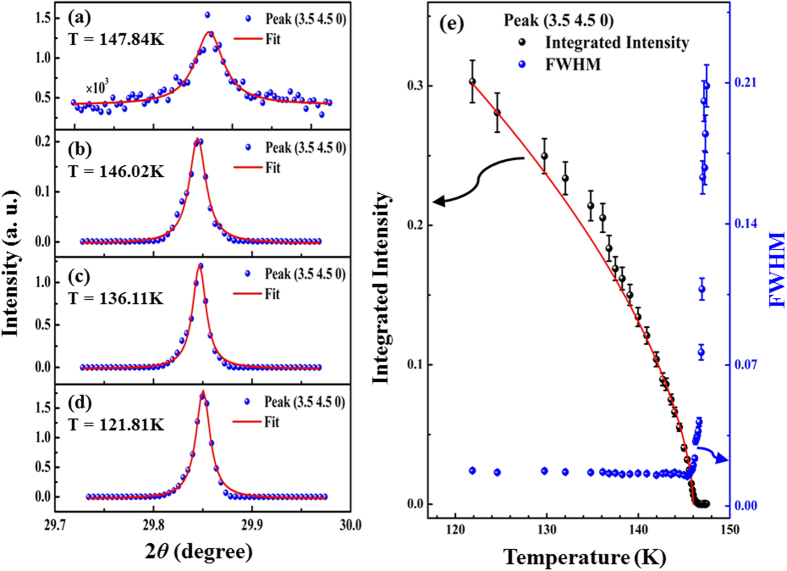
(**a–d)** Evolution of the (3.5, 4.5, 0) peak at various scanned temperatures. The scan temperature is also displayed. Scattered points represent real experimental data, and the continuous solid line is the fitted Lorentz function curve. **(e)** Temperature dependence of integrated intensity and FWHM of (3.5, 4.5, 0) satellite peak. Integrated intensity and FWHM were obtained from the fitting of the peaks. Red solid line is the fitted curve according to the power law.

To investigate a possible CDW modulation in the SIS single crystal, temperature-dependent XRS was conducted. A series of satellite peaks, including (1.5, 1.5, 0), (2.5, 2.5, 0), (3.5, 3.5, 0), and (3.5, 4.5, 0), were observed at temperatures lower than T^*^ (~147 K). The temperature evolution of one of these satellite peaks, (3.5, 4.5, 0), was further studied, as presented in Fig. 2(a–d). The evolution of these satellite peaks at T < T^*^ clearly indicates the distortion of atomic sites and generation of a new ***q***-vector in the direction [*h* ± 0.5, *k* ± 0.5, 0], where *h* and *k* are positive integers. Notably, no such modulated satellite peaks are obtained at temperatures above T^*^. To estimate the integrated intensity and full width at half maximum (FWHM) of the satellite peaks scanned at various temperatures, the peaks were fitted using a Lorentz function [red solid line in Fig. 2(a–d)]. Figure 2(e) plots the variation of the integrated intensity and FWHM with temperature. Significantly, the integrated intensity decreases monotonically as the temperature increases from ~120 K to 147 K, above which it disappears or is insignificant [Fig. 2(e)]. This observation is consistent with the resistivity data, which reveal a transition at a similar temperature, and with the literature on electronically similar CDW materials^21^,^31^. Additionally, the FWHM varies slightly with temperature from ~120 K to 147 K, above which the FWHM increases abruptly. This increase is associated with a very broad and weak satellite peak, suggesting a structural transition. Moreover, the modulated structure develops into a long-range order state but shows divergent behavior above T ~ 147 K, suggesting the characteristics of a second-order transition. The evolution of the order parameter of the modulation, the integrated intensity *I*, as a function of temperature can be fitted to a power law *I* (T)*~* [(T_C_ *-* T)*/*T_C_]^2β^, with 2β~ 0.6 and T_C_ ~ 146.1 K. The value of β is higher than the expected value for 3D systems^32^, and this deviation could be caused by thermal fluctuations. A thermodynamic study has also reported 3D fluctuation behavior for the phase transition that occurs in SIS and SRS compounds^25^. Similar results can be found in the literature on CDW systems^29^,^30^. A recent study of SIS and SRS compounds by C. S. Lue *et al*.^25^ identified strong electron-phonon coupling and a second-order phase transition at T^*^ in SIS and SRS single crystals. The authors observed a trend in the integrated intensity of the (3.5, 4.5, 0) satellite peak with temperature in the SIS system that was similar to that seen in Fig. 2(e). Based on these observations, the (3.5, 4.5, 0) satellite peak is arguably associated with CDW-like instability at the anomalous resistivity transition (~147 K) for T < T^*^^21^,^29^,^30^,^31^.

**Figure 3:**
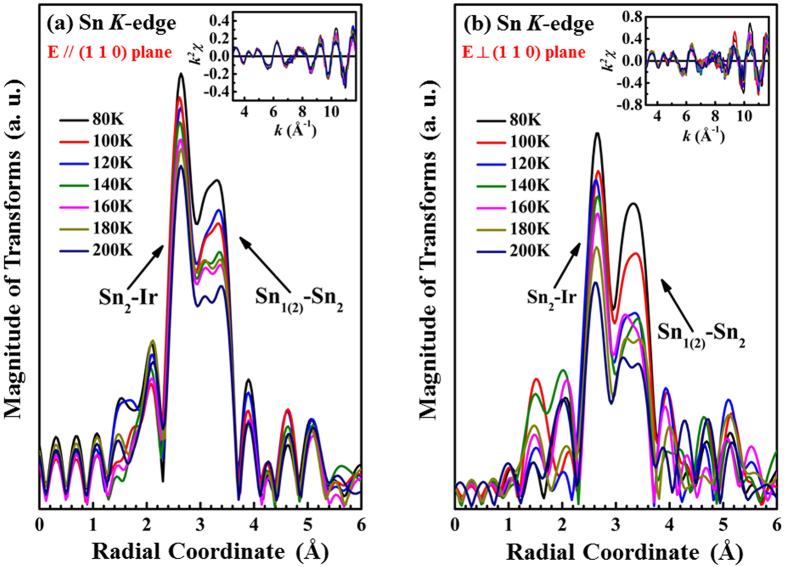
EXAFS spectra of the SIS single crystal at the Sn *K*-edge at various temperatures for the ***E***-field **(a)** parallel and **(b)** perpendicular to the (110) plane. Inset shows the EXAFS *k*^2^χ data, where *k* is in the range from 3 to 11.5 Å^−1^ in all EXAFS spectra.

**Figure 4:**
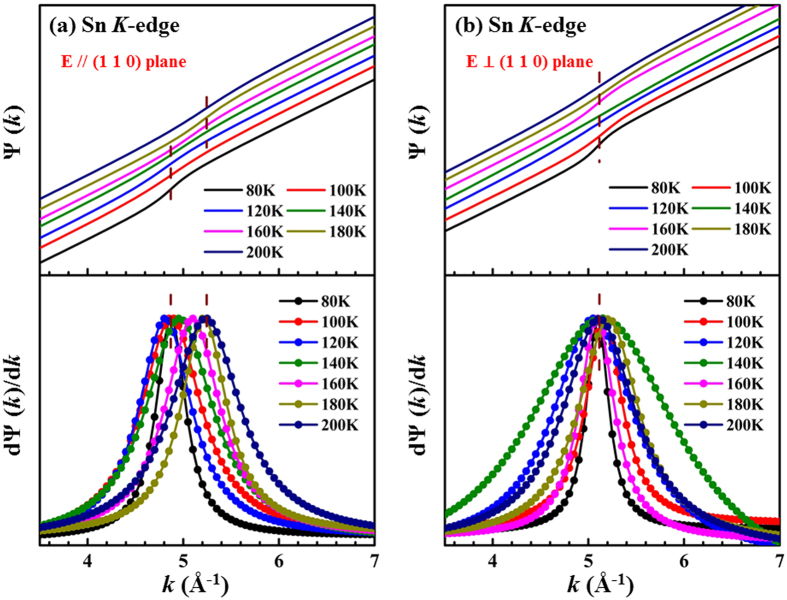
Phase function, Ψ(*k*), and the derivative of the phase function, dΨ(*k*)/d*k*, for the ***E***-field **(a)** parallel and **(b)** perpendicular to the (110) plane. The phase function is extracted from the EXAFS results.

Typically, during the formation of the CDW phase, one or more nuclear magnetic resonance (NMR) split lines are observed because the periodic modulation of the electronic density affects the probed nuclei^20^. Therefore, the lattice distortions in SIS are speculated to have arisen from the CDW instability. Previous studies suggested the splitting of single Sn_2_ atomic sites into four sites at T < T^*^^16^,^20^, so the variation of temperature-induced local atomic structure around the Sn atoms was investigated by performing temperature-dependent EXAFS experiments at the Sn *K*-edge. Figure 3(a,b) present the temperature-dependent Fourier transform (FT) spectra of the Sn *K*-edge with the ***E***-field parallel and perpendicular to the (110) plane, respectively. The insets in Fig. 3(a,b) display the corresponding EXAFS oscillation, χ(*k*), weighted by *k*^2^. The Sn *K*-edge EXAFS spectra exhibit two main FT features at approximately 2.63 Å and 3.34 Å, which correspond to the nearest-neighbor (NN) Sn_2_-Ir (first feature) and the next-neighbor-neighbor (NNN) Sn_1(2)_-Sn_2_ (second feature) bond distances, respectively. As stated above, the Sn subscripts ‘1′ and ‘2′ refer to ‘site 1′ and ‘site 2′ in the complex crystal structure^16^,^20^, respectively. The FT features in Fig. 3(a,b) reveal that the NN Sn_2_-Ir bond distance does not significantly change above *vs*. below the phase transition temperature (the feature position remains almost constant) with the ***E***-field either parallel or perpendicular to the (110) plane. The intensity of the two main FT features increases as the temperature decreases, suggesting that the Debye-Waller (DW) factor decreases as the temperature declines, and the CDW phase transition is primarily related to static disorder, which does not overcome the thermal vibration to enhance the DW factor. Both the thermal vibration and static disorder primarily contribute to the structural disorder of the DW factor. Moreover, the second main feature, NNN Sn_1(2)_-Sn_2_, in the FT spectra with both polarizations is broader than the first main feature, and this difference is the signature of the complex and multiple bond distances around the Sn atoms. The existence of multiple bond distances at low temperature has also been reported in SIS CDW materials^16^,^20^. B. Joseph *et al*.^33^ investigated the IrTe_2_ system using temperature-dependent EXAFS experiments to elucidate the variation of Ir-Te and Ir-Ir bond distances that is associated with the CDW-like phase transition (or an order-disorder transition). To verify the contribution of the distortion at Sn sites in SIS to complex bond distances, the phase function Ψ(*k*) and the phase derivative dΨ(*k*)/d*k* of the Sn *K*-edge EXAFS spectra were obtained and are presented in Fig. 4(a,b) for the ***E***-field parallel and perpendicular to the (110) plane, respectively. This method has been utilized elsewhere to examine the existence of complex bonds^34^,^35^. Ψ(*k*) and dΨ(*k*)/d*k* are commonly obtained to determine the existence of atomic species at tetrahedral and octahedral positions in distorted perovskites. Additionally, this analysis provides accurate information about the associated slightly different bond distances^34^,^35^. The difference between bond distances is obtained via EXAFS *beating point* analysis using the formula


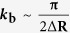


where ∆R is the difference between the two bond distances and ***k***_**b**_ is the *beating point* in the EXAFS oscillation. In the phase derivative analysis, ***k***_**b**_ is extracted from the phase term of the second main peak in the FT spectra; ***k***_**b**_ is associated with NNN Sn_1(2)_-Sn_2_ bond distances with the ***E***-field parallel and perpendicular to the (110) plane, which are shown as dashed lines in the top panels of Fig. 4(a,b), respectively. dΨ(*k*)/d*k* (or ***k***_**b**_) in the lower panel of Fig. 4(a) clearly shows that ***k***_**b**_gradually shifts to a low value as the temperature decreases. This indicates that the difference of the bond distances of Sn_1(2)_-Sn_2_ (∆R) at low temperature (below T = 140 K) exceeds the difference at high temperature (above T = 140 K), according to the above mentioned formula. The lower panel of Fig. 4(a) presents two clear regions separated by a CDW-like phase close to the transition temperature. Below the 140 K peak, the position of dΨ(*k*)/d*k* (or ***k***_**b**_) is shifted to lower *k*, whereas above 140 K, it is shifted to higher *k*, relative to the ***k***_**b**_ of ~5.1 Å^−1^. This result clearly demonstrates the distortion of the Sn sites in the I′ phase. Evidently, the two obtained regions correspond to two non-equivalent bond distances of Sn_1(2)_-Sn_2_. In contrast, the top and lower panels in Fig. 4(b) show the same *k*_b_ of approximately 5.1 Å^−1^, independent of the measuring temperature. This indicates the absence or a subtle distortion when ***E*** is perpendicular to the (110) plane. This phenomenon is not observed in the I′ phase for***E*** perpendicular to the (110) plane. These above mentioned important observations indicate that ∆R of Sn_1(2)_-Sn_2_ in the (110) plane below T^*^ is larger than that above T^*.^ Bond distortion at the Sn sites in the (110) plane is clearly observed and exceeds that perpendicular to the (110) plane at various temperatures. Moreover, broadening of the peak widths of dΨ(*k*)/d*k* with temperature, as shown in the lower panel of Fig. 4(b), is not clear but is possible due to the thermal effect. Electron-phonon couplings in a quasi−1D K_0.3_MoO_3_ CDW material below the CDW phase transition temperature have been found to be greater when the ***E***-field is parallel to the ***b***-axis (in-plane) than when it is perpendicular to the ***b***-axis^26^. In the SIS system herein, at low temperatures, T < T^*^, electron-phonon coupling is observed predominantly along the (110) plane, possibly resulting in the existence of a CDW-like phase in the (110) plane, which is consistent with the temperature-dependent XRS results (Fig. 2). Therefore, the XRS and EXAFS phase derivative analysis clearly demonstrate charge modulation and significant distortion in the lattice of SIS, resulting in the formation of a CDW-like phase at/below T^*^.

**Figure 5:**
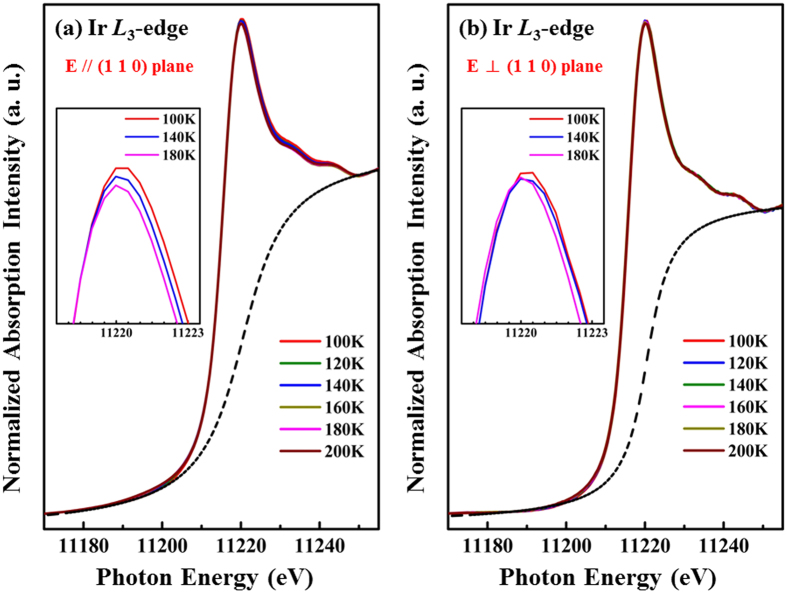
Ir *L*_3_-edge XANES spectra for the ***E***-field **(a)** parallel and **(b)** perpendicular to the (110) plane at various temperatures. Insets in figures display magnified views of the XANES region.

**Figure 6:**
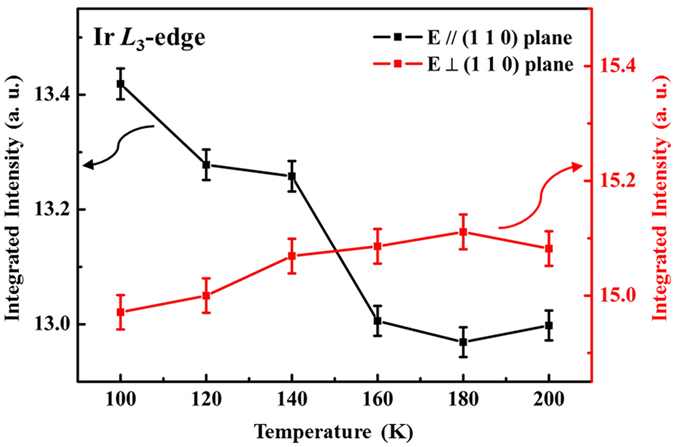
Variation of the integrated intensity of the Ir *L*_3_-edge white-light feature with temperature for the *E*-field parallel and perpendicular to the (110) plane.

**Figure 7:**
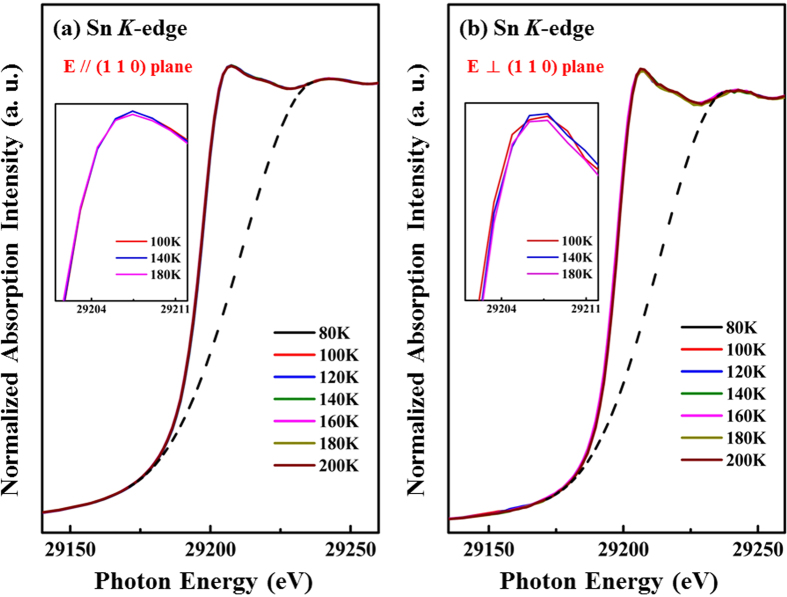
Sn *K*-edge XANES spectra for the ***E***-field **(a)** parallel and **(b)** perpendicular to the (110) plane at various temperatures. Insets in figures display magnified views of the XANES region.

As mentioned above, lattice distortion has been argued to be strongly associated with a CDW transition of the conduction electron system. XANES spectra of the SIS single crystal at the Ir *L*_3_-edge and Sn *K*-edge were obtained to investigate the densities of Ir 5*d* and Sn 5*p* states above the Fermi surface as functions of temperature. Figure 5(a,b) present the Ir *L*_3_-edge XANES spectra with ***E*** parallel and perpendicular to the (110) plane, respectively, at various temperatures. All of these spectra were normalized in the energy range between 11250 eV and 11265 eV (not fully shown in the figures). The dashed lines in the XANES spectra are a best-fitted arctangent function that represents background intensity. According to the dipole-selection rules, the Ir *L*_3_-edge XANES spectra result from the excitations of the Ir 2*p* core states to the unoccupied Ir 5*d*-derived states, and a strong and sharp white-line feature therein indicates a large local density of unoccupied 5*d* states^36^. Interestingly, in Fig. 5(a), the white-line feature intensity increases as the temperature decreases, as clearly seen in the magnified view in the inset. In contrast, the Ir *L*_3_-edge XANES spectra perpendicular to the (110) plane exhibit an almost temperature-independent behavior [Fig. 5(b)], which is also evident in the magnified view in the inset. For clarity, Fig. 6 presents the variations of the integrated intensity of the white-line feature in both directions. The integrated intensity of these white-line features is related to the local density of the unoccupied Ir 5*d*-derived states in the SIS system^26^,^36^. The integrated intensity of Ir 5*d* unoccupied states in the (110) plane remains almost constant from 200 K to 160 K (Fig. 6) and then increases rapidly upon further cooling. This rapid increase in the Ir 5*d* intensity at T < 160 K indicates an increase in the number of Ir 5*d* unoccupied states and an increase in the concentration of holes or *p*-type carriers. In other words, the number of occupied Ir 5*d* electronic states decreases as the temperature falls below 160 K. The temperature independence of integrated intensity is also evident perpendicular to the (110) plane (Fig. 6). Interestingly, the anomaly in the intensity *vs.* temperature profile in the (110) plane occurs at a temperature close to T^*^, which is the temperature of the anomalous resistivity transition in SIS [Fig. 1(a)]. Thus, the anomalous resistivity transition is closely related to the number of Ir 5*d*-derived states. Based on the results of NMR spectroscopy and a study of the temperature-dependent magnetic susceptibility of SIS material by C. N. Kuo *et al*.^20^, the reduction in the number of Ir 5*d* electronic states at T^*^ due to the Fermi surface reconstruction is associated with the anomalous resistivity transition. L. E. Klintberg *et al*.^16^ used the generalized-gradient approximation and local-density approximation calculations to verify the decrease in the number of electronic states near the Fermi surface in the I′ phase. Sn *K*-edge XANES spectra were also obtained with ***E*** parallel and perpendicular to the (110) plane and are displayed in Fig. 7(a,b), respectively. The Sn *K*-edge XANES spectra reflect the transition from the Sn 1 *s* state to the unoccupied 5*p* states^37^. The insets in Fig. 7(a,b) are the magnified views of the near-edge feature at selected temperatures and generally show a temperature-independent behavior, unlike the Ir *L*_3_-edge XANES spectra. No clear variation (or a very subtle increase) in the number of Sn 5*p* unoccupied states with temperature is observed. In the pair potential model, for simple liquid metals, such as Sn and Ga, the distances between rigid point ions exceed the ionic diameters, so the ions behave as inert objects, exhibiting no fluctuation or polarization effects^38^. Therefore, the observation that the behavior of Sn 5*p* electronic states is insensitive to temperature in the Sn *K*-edge XANES spectra is due to the weak fluctuation and core-polarization interaction of the *p*-orbitals of the Sn atoms in the SIS sample^38^,^39^. Thus, from the XANES spectra at the Ir *L*_3_-edge and the Sn *K*-edge, an increase (a decrease) in only the number of unoccupied (occupied) Ir 5*d*-derived states parallel to the (110) plane is observed for T < T^*^. Therefore, an increase in the number of *p*-type carriers in SIS below T^*^, consistent with the Hall effect^25^, further supports the Fermi surface reconstruction of SIS, which is strongly associated with the number of Ir 5*d*-derived states in the CDW-like phase.

In experiments that involve synchrotron radiation, a series of satellite peaks, structural distortion at the Sn sites and a decrease (an increase) in the number of occupied (unoccupied) Ir 5*d*-derived states parallel to the (110) plane were demonstrated at T < T^*^. These results support the CDW modulation in the SIS single crystal at T < T^*^. The possible opening of the band gap at the Fermi surface in the SIS sample was also verified using angle-resolved photoelectron spectroscopy (ARPES) at various temperatures. However, preliminary measurements did not reveal any significant band dispersion and band gap opening at the Fermi surface at/below T^*^. This is probably due to either the fact that the band gap value is significantly smaller than the resolution of the ARPES instrument (~18 meV) or the difficulty of cleaning the sample surface. Certainly, another temperature-dependent ARPES experiment must be performed to further clarify the opening of the band gap of the SIS sample that exhibits the CDW feature, particularly to elucidate how the band gap is related to the nesting properties at the Fermi surface and the *k*-dependent “*kinking*” of the band dispersions that are caused by electron-phonon coupling^40^,^41^. Typically, a perfect or partial nesting of the Fermi surface can induce the CDW and satellite peaks, as observed using XRS in Fig. 2(a) at/below the phase transition temperature. However, the expected opening of the band gap for the CDW was not observed in the SIS system herein. Alternately, this implies that the resistivity and structural transitions of SIS with temperature may not be associated with the conventional CDW but may just involve local Ir 5*d* orbitals. In this case, the crystal-field effect with varying temperature has a prominent role in splitting the conduction electrons in the Ir 5*d e*_g_ and *t*_2g_ levels, reducing the DOSs at/near the Fermi surface. Thus, the electrons and lattice distortions are driven together via free energy minimum. This situation appears to be somewhat similar to the metal-insulator transition in VO_2_^42^,^43^,^44^. Nevertheless, the opening of the band gap of the SIS will be further studied and reported elsewhere later.

In summary, the temperature-dependent XRS, XANES and EXAFS experiments provided evidence of CDW-like instability at the anomalous resistivity transition, T^*^. XRS experiments revealed that the variations of integrated intensity and the FWHM of the satellite peak are similar to those of the established CDW system. EXAFS results revealed the distortion of the arrangement of Sn atoms only in the (110) plane, which is consistent with the XRS results. A decrease (an increase) in the number of occupied (unoccupied) Ir 5*d*-derived states for T < T^*^ and the lack of any significant variation in the number of Sn 5*p* electron states with temperature suggest that the Ir 5*d*-derived states, rather than the Sn 5*p* electron states, have an important role in the anomalous resistivity transition. This study demonstrates significant anisotropy in the electronic and atomic structures of a single crystal of SIS at various temperatures by comparing the XRS, XANES and EXAFS spectra.

## Methods

### SIS single-crystal growth and resistivity measurement

A single crystal of SIS with an area of ~2 mm × 4 mm was grown using the Sn self-flux method^20^,^25^. The crystal normal direction was the [110] plane, as characterized by x-ray diffraction using an in-house x-ray diffractometer with Cu *K*_α1_ radiation. Temperature-dependent resistivity measurements were carried out using a traditional four-point probe technique with probes (four platinum wires) attached to the surface of the (110) plane of the sample. Platinum wires were connected to the surface using silver paste.

### Synchrotron radiation-based measurements

The temperature-dependent Sn *K*-edge XANES, EXAFS, and Ir *L*_3_-edge XANES spectra, with the ***E***-field parallel and perpendicular to the (110) plane, were measured in fluorescence mode using the SWLS-01C and Wiggler-17C beamlines of the National Synchrotron Radiation Research Center (NSRRC), Hsinchu, Taiwan. The energy resolution of the XANES measurements was set to ~0.5 eV (~1.5 eV) at a photon energy of 11.2 keV (29.2 keV), and Ir metal (Sn metal) was used to calibrate the photon energy scale. Temperature-dependent XRS was also performed using an eight-circle diffractometer at the 07 A beamline at NSRRC, Hsinchu, Taiwan. The incident x-ray energy was set to 14.0 keV using a perfect Si (111) double-crystal monochromator. The eight-circle diffractometer allowed the crystallographic axes to be aligned in reciprocal space. To increase the spatial resolution, a high-quality LiF (200) crystal was used as an analyzer. For temperature-dependent measurements, the sample was mounted in a closed-cycle helium cryostat, with a temperature stability of approximately ± 1 K.

## Additional Information

**How to cite this article**: Wang, H.-T. *et al*. Electronic and atomic structures of Sr_3_Ir_4_Sn_13_ single crystal: A possible charge density wave material. *Sci. Rep.* **7**, 40886; doi: 10.1038/srep40886 (2017).

**Publisher's note:** Springer Nature remains neutral with regard to jurisdictional claims in published maps and institutional affiliations.
